# Elementary Physiology for Students

**Published:** 1893-12

**Authors:** 


### Reviews of Books.

*Elementary Physiology for Students.* By ALFRED T. SCHOFIELD, M.D. Pp. xii., 372. London: CASSELL & COMPANY, LIMITED. 1892.

This book is a compilation of the leading facts of animal physiology. A great deal of this difficult science has been compressed into a small volume, and has suffered somewhat in the process. The laudable object of imparting a little interesting knowledge is no doubt fulfilled, and pains have been taken to elucidate the subject. But when we read that "plasma contains egg and serum albumen"; that hæmoglobin belongs to the same class as chondrin and gelatin; that the region between the cortex and medulla is "the seat of the functions of animal life or the soul"; and that "cartilage cells deposit bone," we must confess that such a book cannot be recommended to any earnest student. Many other grave errors are to be found, and the chemistry of the tissues and the physiology of the blood are twenty years behind date. There are numerous diagrams from other books, the source of which is not always acknowledged, and a few original ones. As the science of medicine is built up to a great extent on the rudiments of physiology, inaccuracy in a primer of this nature greatly impairs the usefulness of the work.

